# Osteoclast

**Published:** 1877-05-20

**Authors:** 


OSTEOCLAST.—This is an apparatus invented by C. F. Taylor, M. D., of New York, for fracturing a deformed limb. He reports two cases, wherein disease of the hip joint had resulted in the head of the bone being fixed in the socket, leaving the femur in a distorted and irreducible condition. They were fractured at the distorted point by this instrument treated by fracture apparatus, and the deformity very much improved.

